# *MRPS25* mutations impair mitochondrial translation and cause encephalomyopathy

**DOI:** 10.1093/hmg/ddz093

**Published:** 2019-04-30

**Authors:** Enrico Bugiardini, Alice L Mitchell, Ilaria Dalla Rosa, Hue-Tran Horning-Do, Alan M Pitmann, Olivia V Poole, Janice L Holton, Sachit Shah, Cathy Woodward, Iain Hargreaves, Rosaline Quinlivan, Alexey Amunts, Rudolf J Wiesner, Henry Houlden, Ian J Holt, Michael G Hanna, Robert D S Pitceathly, Antonella Spinazzola

**Affiliations:** 1 MRC Centre for Neuromuscular Diseases, UCL Queen Square Institute of Neurology and National Hospital for Neurology and Neurosurgery, Queen Square, London WC1N 3BG, UK; 2 Department of Clinical and Movement Neurosciences, UCL Queen Square Institute of Neurology, Royal Free Campus, London NW3 2PF, UK; 3 Center for Physiology and Pathophysiology, Institute of Vegetative Physiology, Medical Faculty, University of Köln, 50931 Köln, Germany; 4 Department of Neuromuscular Diseases, UCL Queen Square Institute of Neurology, Queen Square, London WC1N 3BG, UK; 5 Neurogenetic Unit, National Hospital for Neurology and Neurosurgery, Queen Square, London WC1N 3BG, UK; 6 Neurometabolic Unit, National Hospital for Neurology and Neurosurgery, Queen Square, London WC1N 3BG, UK; 7 Science for Life Laboratory, Department of Biochemistry and Biophysics, Stockholm University, 17165 Solna, Sweden; 8 Department of Medical Biochemistry and Biophysics, Karolinska Institutet, 17177 Stockholm, Sweden; 9 Biodonostia Health Research Institute, 20014 San Sebastián, Spain; 10 IKERBASQUE, Basque Foundation for Science, 48013 Bilbao, Spain; 11 CIBERNED (Center for Networked Biomedical Research on Neurodegenerative Diseases, Ministry of Economy and Competitiveness, Institute Carlos III), Madrid, Spain

## Abstract

Mitochondrial disorders are clinically and genetically heterogeneous and are associated with a variety of disease mechanisms. Defects of mitochondrial protein synthesis account for the largest subgroup of disorders manifesting with impaired respiratory chain capacity; yet, only a few have been linked to dysfunction in the protein components of the mitochondrial ribosomes. Here, we report a subject presenting with dyskinetic cerebral palsy and partial agenesis of the corpus callosum, while histochemical and biochemical analyses of skeletal muscle revealed signs of mitochondrial myopathy. Using exome sequencing, we identified a homozygous variant c.215C>T in *MRPS25*, which encodes for a structural component of the 28S small subunit of the mitochondrial ribosome (mS25). The variant segregated with the disease and substitutes a highly conserved proline residue with leucine (p.P72L) that, based on the high-resolution structure of the 28S ribosome, is predicted to compromise inter-protein contacts and destabilize the small subunit. Concordant with the *in silico* analysis, patient’s fibroblasts showed decreased levels of MRPS25 and other components of the 28S subunit. Moreover, assembled 28S subunits were scarce in the fibroblasts with mutant mS25 leading to impaired mitochondrial translation and decreased levels of multiple respiratory chain subunits. Crucially, these abnormalities were rescued by transgenic expression of wild-type *MRPS25* in the mutant fibroblasts. Collectively, our data demonstrate the pathogenicity of the p.P72L variant and identify *MRPS25* mutations as a new cause of mitochondrial translation defect.

## Introduction

Mitochondrial disorders encompass a broad range of pathologies, manifesting as tissue-specific or multisystemic diseases, with onset at any stage of life. Genetically, they can arise from lesions either in the mitochondrial genome (mtDNA) or in nuclear genes required for the maintenance and function of the mitochondria. The advent of next generation sequencing has greatly accelerated the identification of causative disease genes (1). Several of these are involved in the translation of the 13 proteins encoded by the mtDNA that, although few in number, are essential for oxidative phosphorylation (OXPHOS), and thus for energy production. Central to the process of mitochondrial protein synthesis is the mitochondrial ribosome (mitoribosome). In mammals, it comprises a large (mt-LSU, 39S) and a small (mtSSU, 28S) subunit, together sedimenting as 55S particles. The RNA components of the mitoribosome are encoded by mtDNA, whereas the constituent polypeptides (MRPs), some 80 in number, derive from nuclear genes (2). Despite its complexity, defects in the mitoribosome account for only a small minority of mitochondrial translation disorders. To date, mutations in MRPs have been reported for MRPS2, MRPS7, MRPS16, MRPS14, MRPS22, MRPS23, MRPS34, MRPL3, MRPL12 and MRPL44 (3–14). The clinical and biochemical phenotypes vary, but often include neonatal or childhood onset with brain abnormalities, cardiac involvement, increased lactate levels and multiple respiratory chain deficiencies. Here, we report a homozygous *MRPS25* mutation in a patient presenting with mitochondrial encephalomyopathy. Detailed functional analyses supported the pathogenicity of the detected variant and demonstrate the important role for MRPS25 in mitochondrial ribosome function.

## Results

### Case report

**Figure 1:**
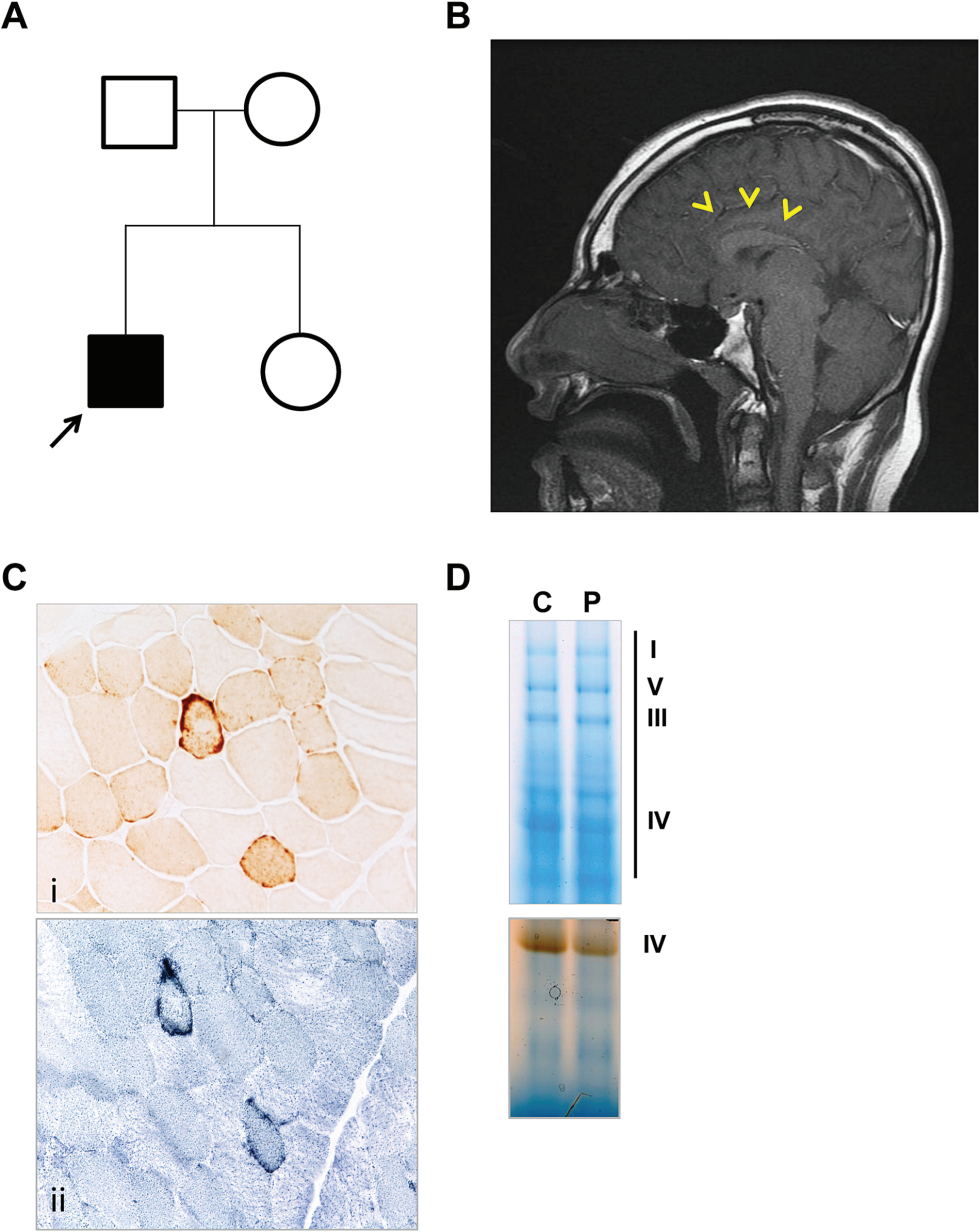
Neuroradiological and histological findings. (**A**) Pedigree of the family; the proband is indicated by the black arrow. (**B**) T1 sagittal brain MRI showing partial agenesis of corpus callosum (yellow arrowheads). (**C**) Cytochrome *c* oxidase staining (i) reveals a mild and generalized decreased activity with rare fibres with subsarcolemmal mitochondrial aggregates typical of ‘ragged-red’ alterations (succinate dehydrogenase, ii). (**D**) Complex IV in-gel activity is decreased in the proband’s muscle sample.

The proband is a 25-year-old male, first child of healthy non-consanguineous parents (Fig. 1A). He was born at term with a birth weight of 2.5 kg following a normal pregnancy, although at 28 weeks intra-uterine growth restriction was detected. After an uneventful perinatal period, psychomotor delay became evident and, at 8 months his weight was 6.34 kg (beneath the 3rd centile) and head circumference was 40 cm (beneath the 3rd centile). At 10 months, he exhibited poor head control, choreoathetoid distal limb movements associated with increased extensor tone and brisk reflexes. A brain magnetic resonance imaging (MRI) scan at this time revealed partial agenesis of corpus callosum and under-development of the frontal and parietal temporal regions, while extensive investigation for an underlying metabolic disorder, including measurement of mucopolysaccharides and oligosaccharides, serum copper level, white cell enzymes, very long chain fatty acids, carnitine levels, pyruvate dehydrogenase activity and urine organic acid, failed to detect any abnormalities. Karyotyping and echocardiogram were normal too, while blood and CSF lactate showed borderline values (2.8 mmol/l; 2.7 mmol/l, respectively, normal values < 2.1 mmol/l). Histochemical and biochemical analyses of skeletal muscle were unremarkable, with the exception of a mild increase in lipid content in electron microscope images. Motor milestones were subsequently delayed: he rolled at 18 months, crawled at 48 months and could only walk a short distance unaided at 9 years of age. His mobility performance was further compromised by the development of hip dysplasia at 11 years, for which surgery was required at 16 years, following which mobility was reduced to a wheelchair. In the meantime, when he was 10 years old, he was diagnosed with adrenal insufficiency and required hydrocortisone replacement. Since this time, the clinical course has been progressive, with gradual worsening of muscle fatigue and weakness and dysphagia. When reviewed in our specialist mitochondrial clinic at 18 years of age, he had short stature (150 cm) and microcephaly (head circumference of 48.3 cm) and was alert and able to communicate using a communication device. In the limbs, there was generalized dystonia with global reduction in strength, symmetrically brisk reflexes and downgoing plantar responses. Brain MRI confirmed partial agenesis of the corpus callosum (Fig. 1B) and a repeat of the metabolic investigations was normal, except for a mild elevation of the plasma lactate (2.14 mmol/l; normal value, 2.1). A second muscle biopsy aged 19 years showed a uniform decrease of cytochrome c oxidase (COX) histochemical staining, and complex IV deficiency measured by spectrophotometric analysis (0.007 Complex IV/Citrate Synthase ratio; normal values, 0.014–0.034) and in-gel enzyme activity (Fig. 1C and D). Enzyme assays of complex I and II + III were within the normal range.

### Exome sequencing identified a homozygous mutation in *MRPS25* in the proband

With the hypothesis of a mitochondrial aetiology of the disorder, mtDNA was studied in patient samples (blood and muscle). The analysis excluded pathogenic point mutations, rearrangements or decreased mtDNA copy number (data not shown); therefore, whole exome sequencing was undertaken in both the proband and his parents.

**Figure 2:**
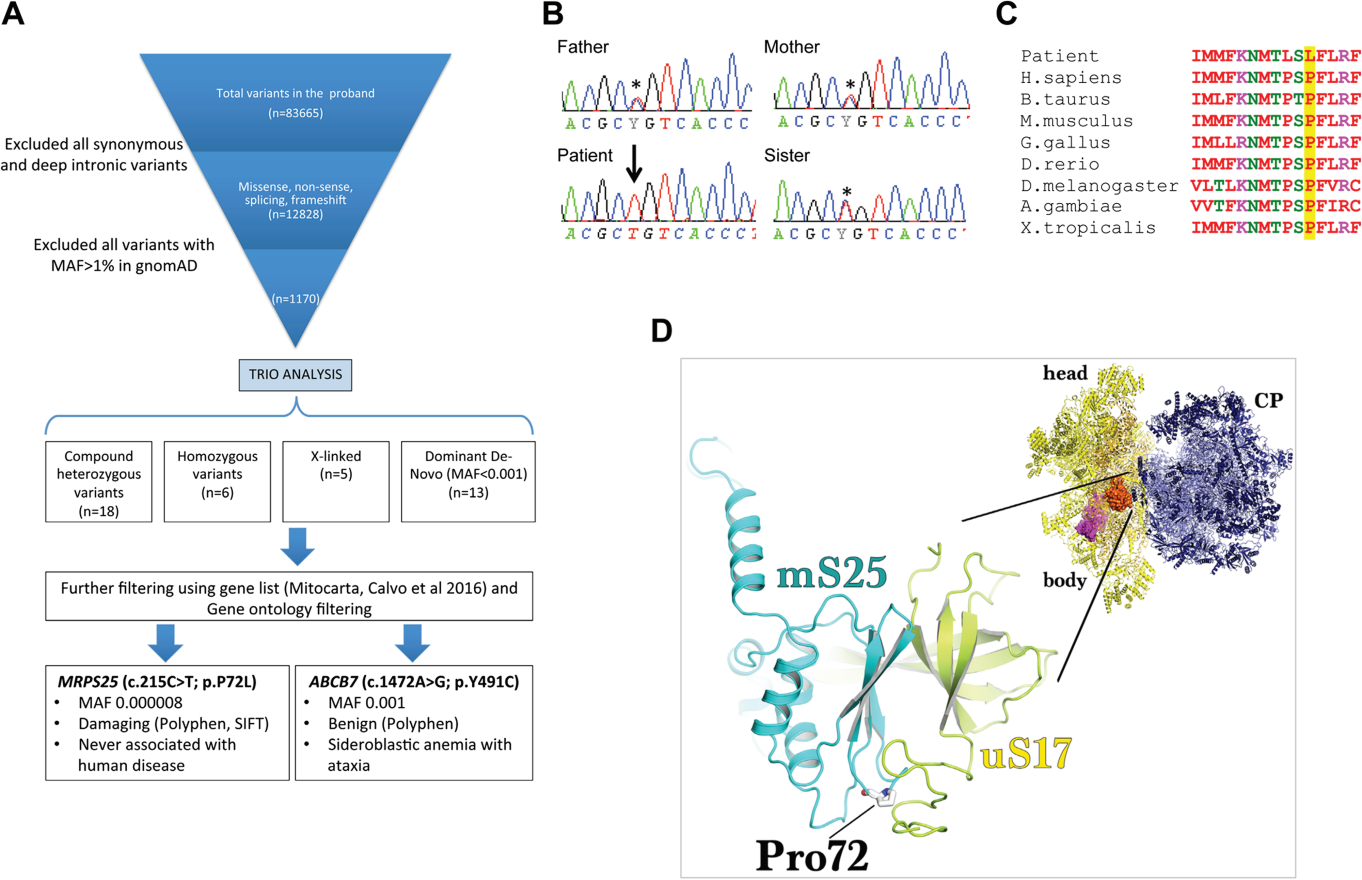
Identification of a potential pathogenic *MRPS25* mutation by exome sequencing. (**A**) Filtering of the identified variants was performed using Varaft platform by comparing the proband’s and parents’ exomes. The analysis resulted in 42 genetic variants (*de novo*, recessive and X-linked), of which two were in genes encoding for mitochondrial proteins. Only the c.215C>T change in *MRPS25* was predicting as damaging (using SIFT and Polyphen tools). (**B**) Sanger sequencing confirmed that each parent and the healthy sister were heterozygous (asterisk) for the mutation in *MRPS25* while the affected patient was homozygous (arrow). (**C**) Sequencing alignment of the human MRPS25 protein shows the evolutionary conservation of the Pro72. (**D**) The modelling of Pro72Leu (stick representation) mutation on the structure of the human mitoribosome (PDBID: 3J9M) reveals that it is likely to sterically hinder the formation of interprotein contacts with uS17 (yellow) as well as result in a potential destabilisation of the folding of the essential strand-turn-strand in the mS25 (cyan) protein core. The inset of the mitoribosomal structure illustrates the relative position of uS17-mS25 (surface representation) on the small subunit (yellow cartoon); CP, central protuberance.

A series of filtering steps (using Varaft platform (15)) were applied to the identified variants and Trio-based analysis performed, considering recessive, de-novo dominant and X-linked inheritance pattern (Fig. 2A). A list of rare genetic variants was generated (Supplementary Material, Table S1), but none of the variants were consistent with the proband’s phenotype, including the agenesis of the corpus callosum. Further filtering against nuclear genes encoding mitochondrial proteins yielded two candidate genes: *ABCB7* and *MRPS25*, encoding an ABC transporter and a structural subunit of the mitochondrial ribosome, respectively.

The variant in *ABCB7* (c.1472A>G; p.Y491C) on chromosome X has a high frequency in the population (Minor allele frequency, MAF = 0.001) and is predicted to be benign by multiple *in silico* tools (Sorting Intolerant From Tolerant, SIFT 0.058, tolerate, Polyphen2 HDIV 0.028, Benign; Polyphen2 HVAR 0.088, Benign). In contrast, the homozygous nucleotide change in *MRPS25*, c.215C>T; p.P72L, is extremely rare in the general population (MAF 0.000008 in Genome Aggregation Database, gnomAD, August 2018; absent in Exome variant server (ESP6500)), and no homozygous cases are listed. Subsequent Sanger sequencing of the family members confirmed the variant to be homozygous in the proband’s DNA, whereas both parents and the healthy sister were heterozygous carriers for the nucleotide change (Fig. 2B). The p.P72 residue is highly conserved (Fig. 2C), and its substitution by leucine is predicted to be damaging (SIFT score = 0.03; and PolyPhen-2 score = 1 for both HVAR and HDVI). The three-dimensional structure of the human ribosome (16) places mS25 on the solvent side of the 28S subunit. Structural modelling suggests that the P72L substitution is likely to sterically hinder inter-protein contacts, particularly those with uS17 (Fig. 2D), as well as resulting in a secondary destabilisation of the folding of the essential strand-turn-strand in the mS25 protein core. The latter potentially compromises the assembly or stability of the 28S mitochondrial ribosomal subunit. Thus, both *in silico* analysis and modelling predict a deleterious effect of the p.P72L substitution.

**Figure 3:**
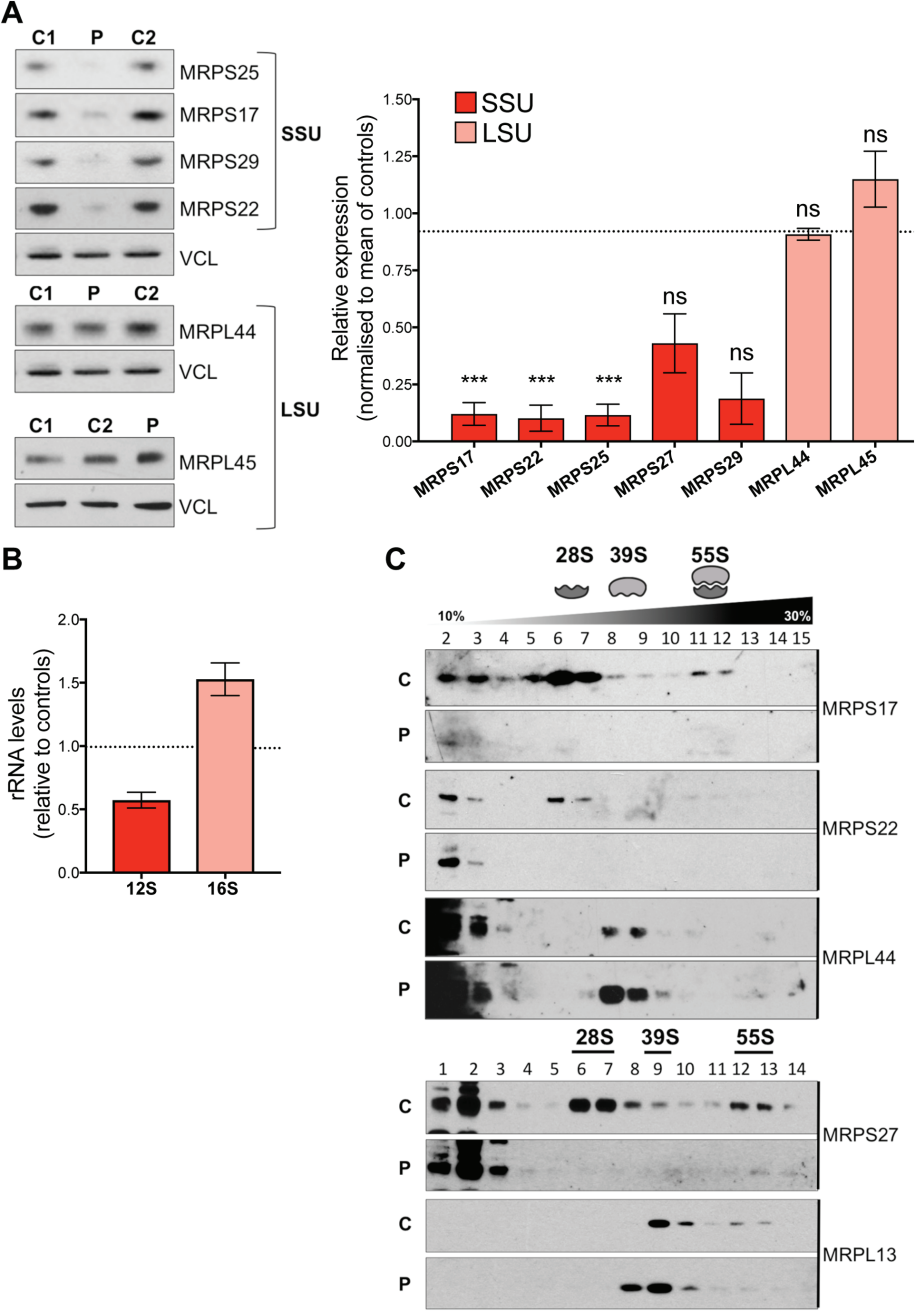
*MRPS25* mutation compromises the maintenance of normal 28S subunit levels. (**A**) Steady state levels of small (MRPS25, MRPS17, MRPS29 and MRPS22) and large (MRPL45, MRPL44) mitoribosomal subunits in controls (C1 and C2) and patient (P) total fibroblast extracts. Vinculin (VCL) is shown as a loading control. The mean relative abundances are shown to the right based on Fiji ImageJ densitometric analysis. Data are expressed as mean ± standard error of the mean of *n* = 3 independent experiments, except MRPL27 and MRPS29 (*n* = 2). Probability was determined using Welch’s *t*-test (ns = not significant, *P* > 0.05; ^*^P < 0.05; ^***^P < 0.001). (**B**) Relative 12S and 16S rRNA levels in patient-derived fibroblasts. The data are representative of two independent experiments. (**C**) Isokinetic sucrose gradients (10–30%) were used to analyze the distribution of mitochondrial ribosome in total lysates from control (C) and patient (P) fibroblasts. Mitochondrial ribosomal protein markers of the mt-SSU (MRPS27 and MRPS22) and the mt-LSU (MRPL13 and MRPL44) ribosomal subunits were detected by immunoblotting. The data are representative of three independent experiments.

### MRPS25*-*P72L is associated with low levels of MRPS25 protein and its 28S subunit partners

To investigate the effect of the mutation on the MRPS25 protein levels we performed immunoblot analysis on fibroblast extracts from the subject and two controls. The steady-state level of MRPS25 in the proband’s fibroblasts was approximately one-tenth of the level of the controls (Fig. 3A), indicating the mutation causes MRPS25 instability. Since MRPS25 is a structural subunit of the 55S ribosome, we investigated whether other MRPS components were affected by the lack of MRPS25. The abundance of three other polypeptides of the 28S subunit, MRPS17, 22 and 29, was severely affected in the subject’s fibroblasts, while the two components of the large mitochondrial ribosomal subunit (MRPL44 and 45) analysed were not decreased (Fig. 3A). The same contrast between the large and small ribosomal subunits was evident in the levels of the ribosomal RNAs: the 12S rRNA component of the 28S subunit was 60% of the control value, while the 16S rRNA of the 39S subunit was 1.5 times the control value (Fig. 3B). Moreover, analysis of the mitochondrial ribosome profile on sucrose gradients showed that, in the patient fibroblasts, there was very little intact 28S subunit, and the residual MRPS components were concentrated at the top of the gradient among soluble and individual polypeptides, whereas the 39S subunit was overall normal, albeit a small fraction distributed at a lower buoyant density than controls (Fig. 3C, see also the outcome of complementation, Fig. 6). Together, these data indicate that the identified mutation principally affects the assembly or stability of the small ribosomal subunits.

### Mutant fibroblasts show impaired mitochondrial protein synthesis and multiple respiratory chain defects

**Figure 4:**
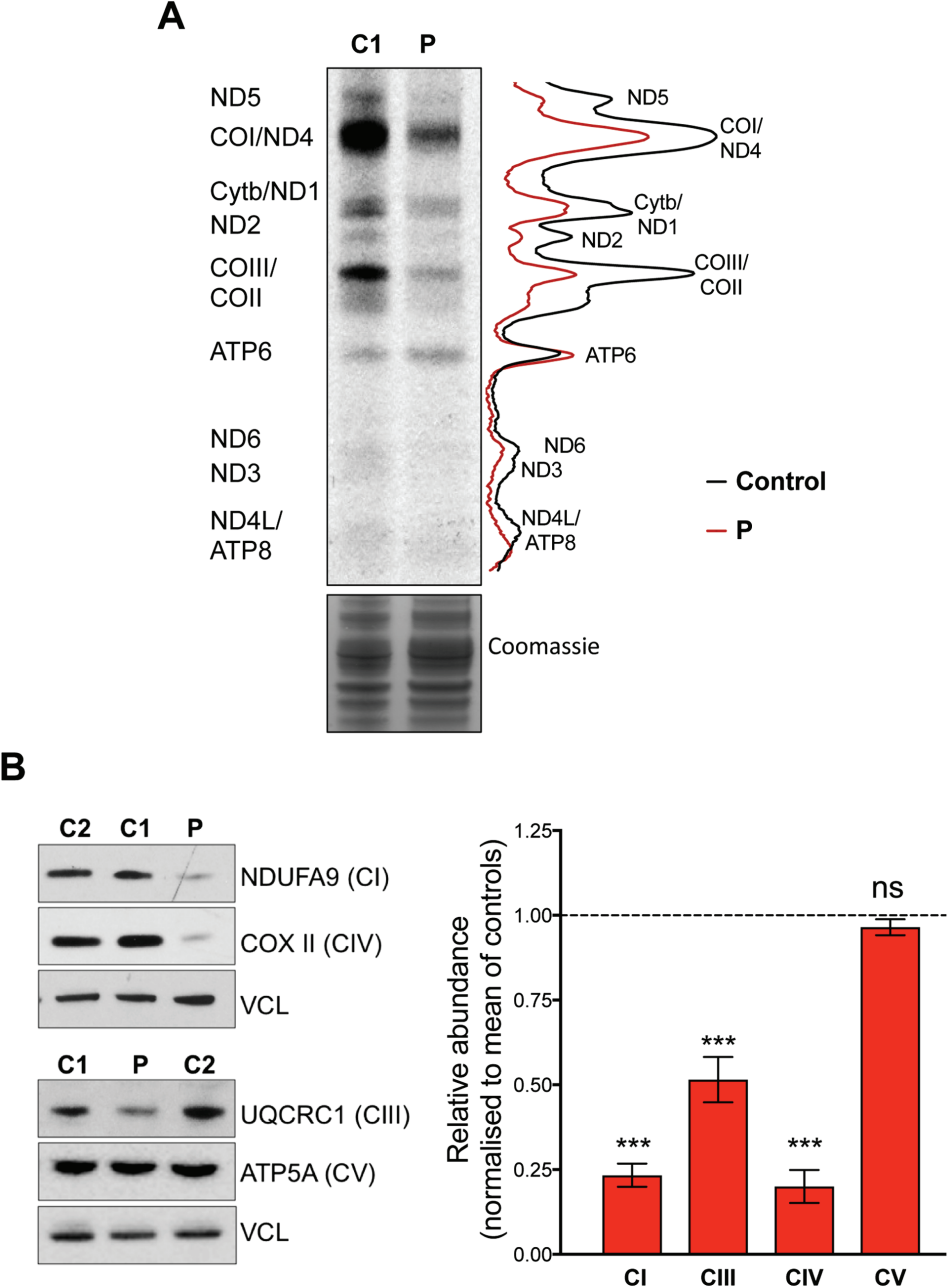
*MRPS25* mutation reduced mitochondrial translation and causes a combined OXPHOS defects in the affected patient. (**A**) *De novo* mitochondrial protein synthesis measured by ^35^S-methionine incorporation in control (C1) and patient (P) fibroblasts. The gel image is flanked by polypeptide assignments to the left and plot profiles showing the pixel intensities for the control (black line) and patient (P–red line) are shown on the right. Coomassie staining of total protein was used as loading control. (**B**) To the left, representative immunoblots of OXPHOS components of complex I (NDUFA9), complex III (UQCRC1), complex IV (COXII) and complex V (ATP5A). Levels of vinculin (VCL) were used as indicators of protein loading. To the right, a chart indicating the abundance of the respiratory chain proteins in the patient fibroblasts compared to controls (Fiji ImageJ densitometric analysis). The data are the mean ± standard error of the mean of *n* ≥ 3 independent experiments. Probability was determined using Welch’s *t*-test (ns = not significant, *P* > 0.05; ^***^P < 0.001).

The scarcity of the mitoribosomes implies a reduced mitochondrial translation capacity that is expected to limit the production of the mtDNA-encoded proteins required for the OXPHOS system. Radiochemical labelling of newly synthesized mitochondrial proteins demonstrated that mitochondrial translation was compromised in cells with MRPS25-P72L (Fig. 4A). The impaired translation was accompanied by decreases in the steady-state levels of proteins of respiratory chain complexes I, III and IV (Fig. 4B), with complex III being the least affected of the proton-translocating enzymes of the respiratory chain (Fig. 4A and B). In contrast, ATP synthase subunit 6 was translated in amounts similar to control cells, suggesting that it takes priority. These findings indicate that MRPS25 is required for mitochondrial protein synthesis and that the impairment in translation in the mutant fibroblasts results in decreased levels of the mitochondrial respiratory chain subunits.

### Delivery of wild-type *MRPS25* rescues the mitochondrial ribosomal assembly and OXPHOS protein levels

**Figure 5:**
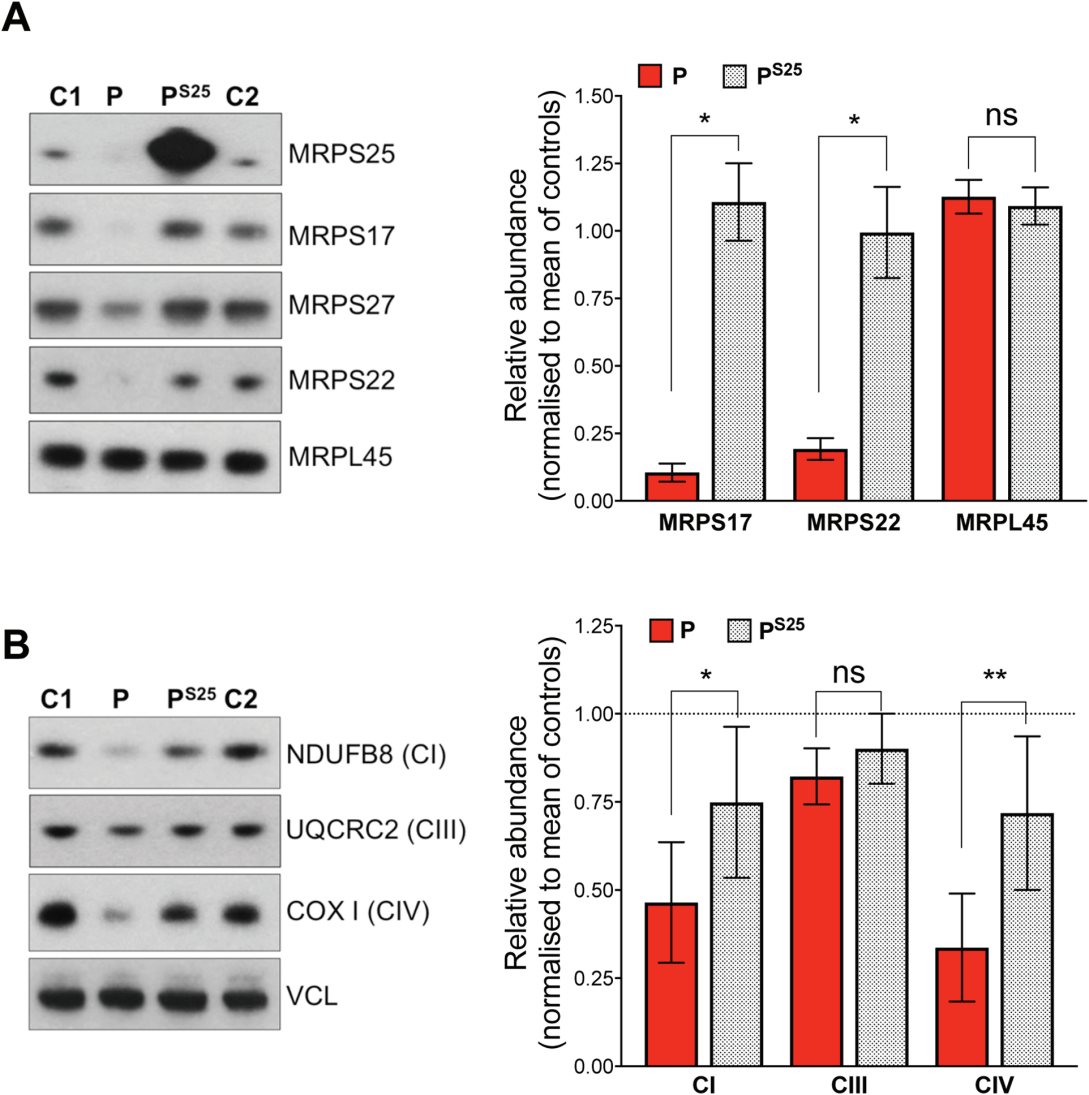
Lentivirus mediated expression of wild-type MRPS25 rescues the mt-SSU and OXPHOS levels in patient cells. Analysis of the small (MRPS25, MRPS17, MRPS27, MRPS22) and large (MRPL45) mitochondrial subunits (**A**) and OXPHOS components of complex I (NDUFB8), complex III (UQCRC2), complex IV (COXI) (**B**) in immortalized cell extracts from control (C1 and C2), patient (P) and patient transduced with wild-type *MRPS25* (P^S25^). Vinculin (VCL) was used as a loading control. To the right of A and B, relative abundance of the ribosomal protein and OXPHOS components in untransduced versus the transduced fibroblasts (Fiji ImageJ densitometric analysis). The data are shown as the mean ± standard error of the means of *n* ≥ 3 independent experiments. Probability was determined using Welch’s *t*-test (ns = not significant, *P* > 0.05; ^*^P < 0.05).

**Figure 6:**
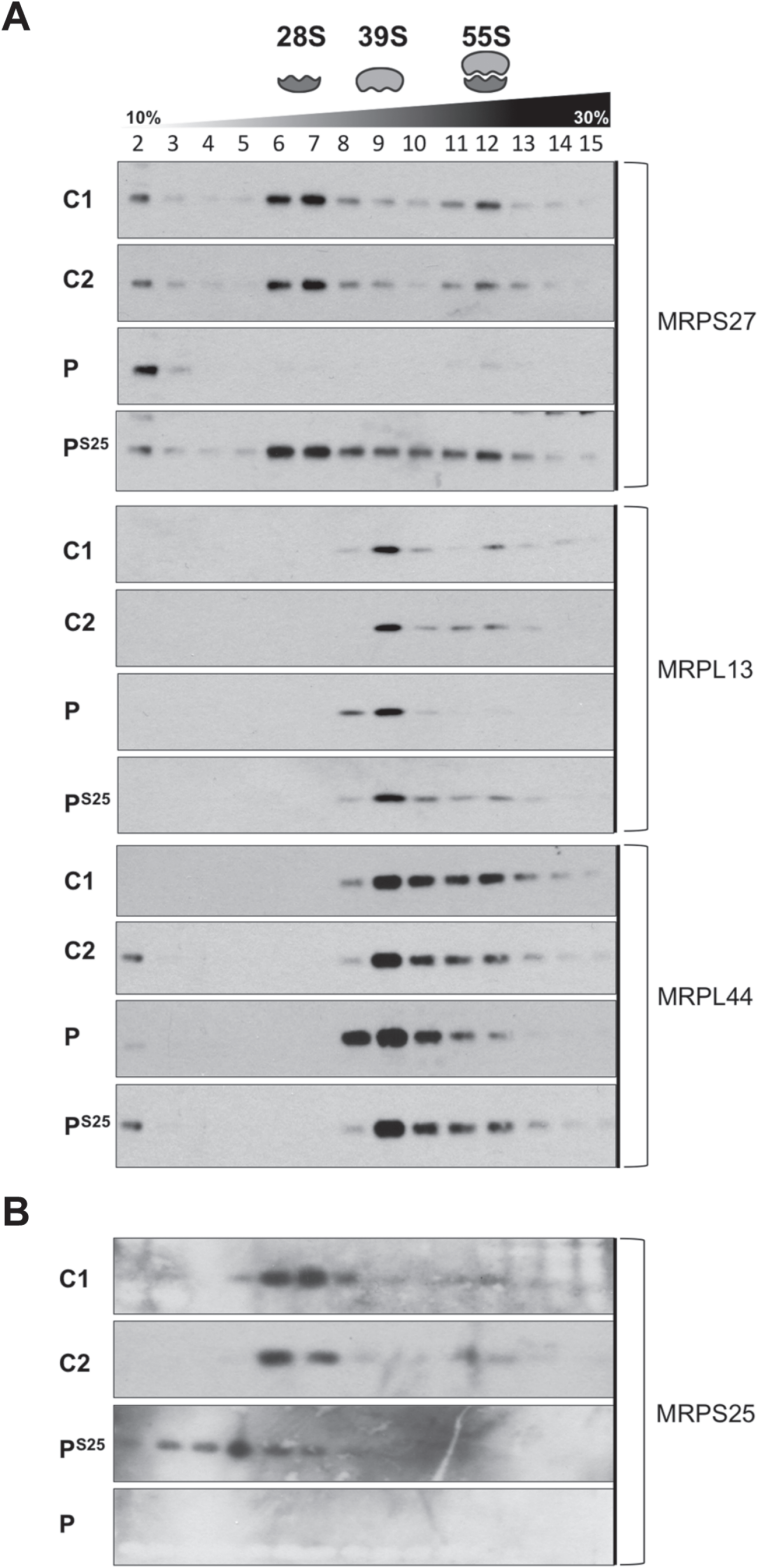
Rescue of the mitoribosomes assembly in patient cells transduced with wild-type *MRPS25*. (**A**) Mitochondrial ribosomes from controls (C1 and C2) and patient without (P) and with (P^S25^) wild-type *MRPS25* cDNA were sedimented on sucrose gradient and fractions separated by SDS-PAGE. Mitochondrial ribosomal protein markers of the mt-SSU (MRPS27) and the mt-LSU (MRPL13 and MRPL44) were detected by immunoblotting. The data are representative of two independent experiments. (**B**) As in (A), except that MRPS25 protein distribution was analysed.

To further validate the pathogenicity of the *MRPS25* c.215C>T; p.P72L variant, we performed complementation studies by introducing wild-type *MRPS25* into immortalized fibroblasts. Lentiviral-mediated expression of *MRPS25* in control cells resulted in marked cell death, suggesting a possible toxic effect of the protein in a context of normal assembly of the 28S subunit. In contrast to the controls, transgenic MRPS25 was well tolerated in the cells with the MRPS25-P72L variant, where it resulted in the restoration of MRPS levels (Fig. 5A) and the 28S subunit (Fig. 6), accompanied by partial restoration of OXPHOS protein levels (Fig. 5B). Collectively, these data establish the disease-causing nature of the *MRPS25* mutation.

## Discussion

Defects in mitochondrial translation are among the earliest known causes of mitochondrial disease, as many result from mutations in the transfer RNA genes that are encoded in mtDNA (17). However, over the course of three decades, pathological mutations in many components of the mitochondrial protein synthesis machinery have been identified, all of which are encoded in nuclear DNA (18). Notwithstanding this, deleterious mutations among the 80 structural proteins of the mitoribosome are still rare, numbering around 10 MRP genes; moreover, each of these accounts for no more than a handful of patients worldwide. The current study adds MRPS25 to the set of MRPs whose loss of function causes human disease. Several lines of evidence support the pathogenicity of the p.P72L variant: first, the mutation results in a marked reduction of the MRPS25 protein (Fig. 3A); second, the lack of MRPS25 appears to destabilize the entire small ribosomal subunit, which consequently limits the rate of translation (Figs 3B and 4A), and OXPHOS protein levels (Fig. 4B); finally, complementation of the proband’s fibroblasts with wild-type *MRPS25* restored the 28S subunit and increased the levels of OXPHOS proteins (Figs 5 and 6).

MRPS25 is one of 15 structural subunits of the mitochondrial 28S that does not have a bacterial homolog (19). It is therefore the result of the process by which the mitochondrial ribosomes have diverged from their bacterial origin by reducing their RNA content and increasing the number of proteins. The functional role of MRPS25 has not previously been investigated; our current analysis indicates that MRPS25 is essential to the assembly or stability of the 28S subunit.

While it was intuitive that a damaging mutation in MRPS25 would alter the 28S subunit, less expected was the effect on the 39S subunit. In addition to the increase in the 16S rRNA (Fig. 3B), there was a modest shift in the distribution of the 39S subunits (Fig. 3C). That this represented a genuine effect of the mutant was corroborated by the analysis of the mutant fibroblasts complemented with wild-type *MRPS25*, where the distribution of the 39S subunits on the sucrose gradient matched the profile of control cells (Fig. 6A). Revisiting the literature, we found that other studies have observed alterations to the 39S subunit stemming from defects in 28S subunit components, although these were not always discussed. In transgenic mice mutated for *MRPS34*, the abundance of large subunits was increased in young animals, and declined in aged tissues, associated with alterations of the buoyant density of a fraction of the 39S subunit (20). Likewise, complexomic profiling revealed increased abundance of the 39S subunit and small changes in its mobility (complex size) in fibroblasts carrying mutant MRPS2 (3). In addition, as in our study, a redistribution of the large subunit on sucrose gradients was observed in fibroblasts with mutant MRPS34 (9). These alterations to the 39S subunit may reflect abortive attempts to form the 55S ribosome, or be the result of unstable 55S subunits breaking apart owing to the distortions created by the mutant MRPS25.

**Figure 7:**
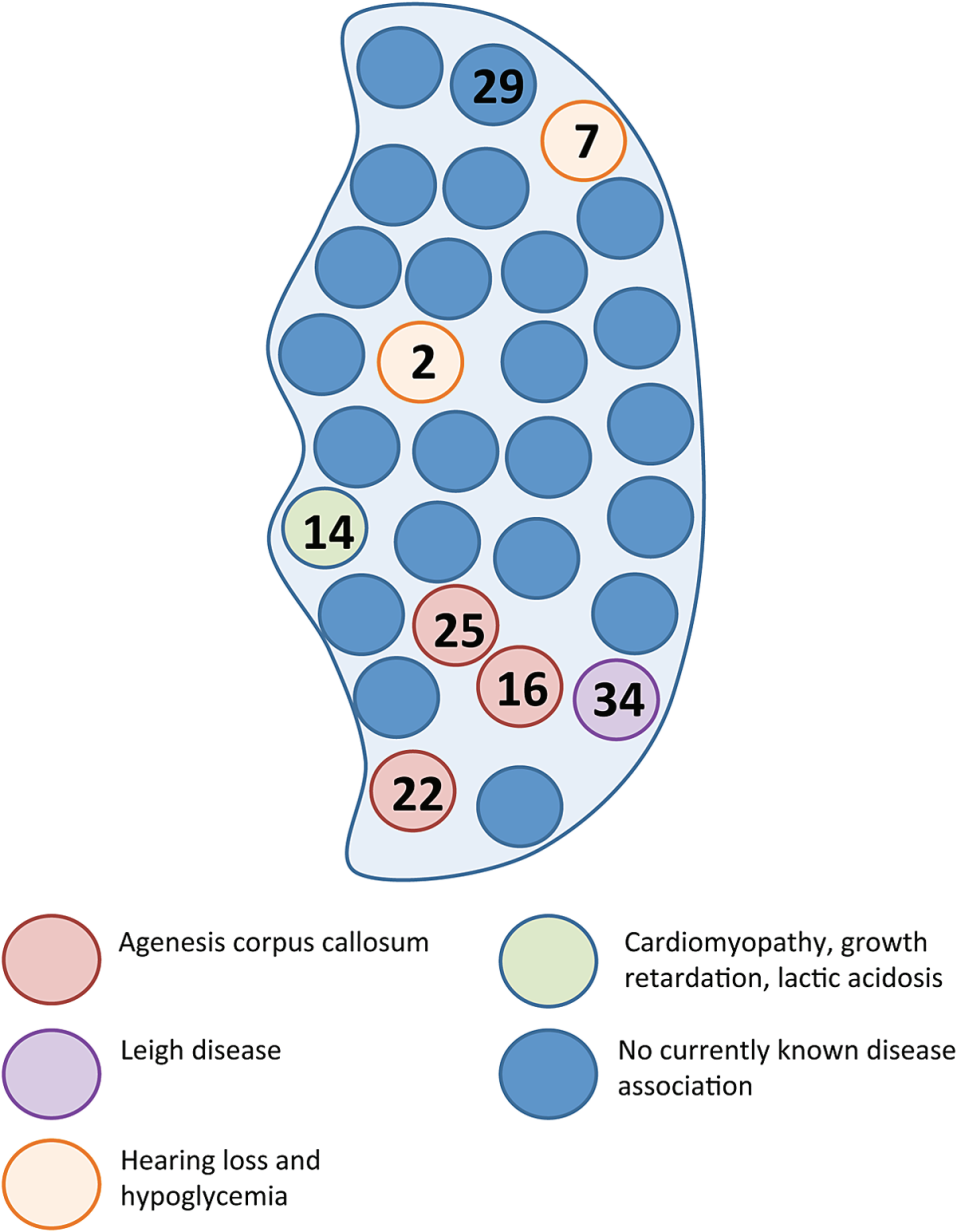
The 28S subunit and mitochondrial disease. Schematic representation of the mitochondrial 28S subunit with circles representing each of the 30 constituent proteins. Numbers in the circles are specific MRPS associated with mitochondrial disease. Defects in MRPS16, MRPS22 and MRPS25, closely juxtaposed in the 28S subunit, are associated with agenesis of corpus callosum.

As with many other mitochondrial diseases, defects in mitochondrial translation cause an extremely broad spectrum of symptoms and signs. Nevertheless, there are some consistent features among the known mutations in the mitoribosome proteins. The pathological mutation in *MRPS25* reported here adds to those in *MRPS16* and *MRPS22* that are also associated with defects of the corpus callosum, which, in the case of the *MRPS25* mutant, was partial. Thus, defects in the small subunit of the mitochondrial ribosome should be considered when defects of the corpus callosum are present. All three polypeptides are closely juxtaposed in the 28S subunit (Fig. 7), although mutations in MRPS34, immediately adjacent to MRPS16, result in Leigh or Leigh-like syndrome (9).

Overall, brain involvement (structural or functional) is common among disorders that arise from mutations in MRPs; in addition to defects of the corpus callosum and Leigh syndrome, dilatation of cerebral ventricles and microcephaly have been reported (7). Cardiomyopathy may also result from mutations in MRPS genes (7), although it was not evident in our case.

In conclusion, MRP mutants are a rare but rapidly emerging group of mitochondrial translation disorders, which exhibit considerable clinical heterogeneity. Although a whole exome or genome sequencing approach is frequently required to confirm the diagnosis, early-onset multi-system disease associated with structural brain disease on MRI should prompt interrogation of this subset of genes. Moreover, our study indicates that defects in the translation machinery can result in isolated complex IV deficiency in muscle, rather than in dysfunction of multiple respiratory chain enzymes. This observation suggests that dedicated mitochondrial ribosomal proteins regulate the translation of specific respiratory chain subunits in mammals, as recently proposed for yeasts (25), providing an explanation of the different mitochondrial ribosomal profiles among tissues (26), and the tissue specific manifestations of MRP-related diseases.

## Materials and Methods

Samples from the proband and family members were obtained after receiving written informed consent. The study was performed in accordance with the Declaration of Helsinki and approved by our institutional review board.

### Histochemical and biochemical studies in skeletal muscle

Analyses of muscle morphology, histochemistry and respiratory chain activities were performed as previously described (21,22).

### Genetic analysis

DNA was extracted from muscle and peripheral leukocytes by standard methods. For mtDNA sequencing, 45 overlapping fragments were amplified and Sanger sequenced using BigDye Terminator v1.1. Bi-directional sequencing was run on an ABI 3730xl. Data were assembled and analysed by SeqScape v3 using the revised Cambridge Reference sequence NC_012920.1 for alignment.

The assessment of large-scale rearrangements of mtDNA, both single and multiple deletions in muscle, was performed by long-range polymerase chain reaction (PCR).

Whole exome sequencing was performed on the proband and parents. The samples were prepared according to an Agilent SureSelect Target Enrichment Kit preparation guide. The libraries were sequenced with HiSeq 2000/2500 sequencer (Illumina). Sequencing data were aligned to the human reference genome, hg19 (GRCh37; UCSC genome browser), using Burrows–Wheeler Alignment Tool and variants were called using GATK. Filtering of variants was performed using VarAft platform (http://varaft.eu/). We filtered out variants with allele frequency higher than 1% in gnomAD, 1000 Genome or ESP6500 database, synonymous and deep intronic variants. Finally, we prioritized variants reported in genes listed in mitocarta (23) and variants associated with the phenotype under study.

### Cell culture

Primary skin fibroblast cultures were obtained from healthy controls and the patient and confirmed free of mycoplasma based on the LookOut Mycoplasma PCR Detection Kit (Sigma). Primary fibroblasts were cultured in Dulbecco’s Modified Eagle’s Medium (DMEM, LifeTechnologies) supplemented with 10% fetal bovine serum (FBS, Hyclone), 1% penicillin and streptomycin (PS, Life Technologies) at 37°C in a 5% CO_2_ atmosphere.

### Western blotting and immunodetection

For western blotting, cells were lysed in phosphate buffered saline (PBS) solution containing 1% SDS, 0.1% *n-*dodecyl-D-maltoside (DDM), 1X protease inhibitor cocktail (Roche), phosphatase inhibitor cocktail (Abcam) and 50 Units Benzonase (Millipore). Protein concentration was determined by DC protein assay kit (Biorad). Protein samples were prepared in 1× Laemmli loading buffer, heated at 42°C for 15 min and resolved on SDS-PAGE gels (Novex, Thermofisher Scientific). After electrophoresis, proteins were transferred to polyvinylidene fluoride membranes (PVDF, Millipore). Membranes were blocked with 5% non-fat dry milk in PBS with 0.1% (v/v) Tween-20 (PBST) and incubated overnight at 4°C with the primary antibodies listed in Supplementary Material, Table S2. Membranes were then washed three times with PBST and incubated with secondary antibodies conjugated with horseradish peroxidase (HRP) (Promega) at 1:5000 dilution in 5% milk PBST. After three washes, immunoblots were developed using enhanced chemiluminescence (ECL Prime, GE Healthcare).

### [^35^S]-methionine cell labelling for mitochondrial protein synthesis

Mitochondrial translation products were labelled using ^35^S-methionine as previously described (24). Fibroblasts were washed twice with methionine/cysteine free DMEM (Life Technologies) supplemented with 1 mM L-glutamax, 96 μg/ml cysteine (Sigma), 1 mM pyruvate and 5% (v/v) dialyzed FBS, and incubated in the same medium for 10 min at 37°C. A total of 100 μg/ml emetine dihydrochloride (Sigma) was then added to inhibit cytosolic translation, before pulse-labelling with 100 μCi [^35^S]-methionine for 45–60 min. Cells were chased for 10 min at 37°C in regular DMEM with 10% FBS, washed three times with PBS and collected. Labelled cells were lysed in PBS, 0.1% *n-*dodecyl-D-maltoside (DDM), 1% SDS, 50 units Benzonase (Millipore), 1X protease inhibitor cocktail (Roche). Protein concentration was measured by DC protein assay kit (Biorad) and 20 μg of protein were separated by 12% SDS-PAGE. Gel were fixed and vacuum dried, and exposed to X-ray film for 1 week.

### RNA quantification

Total RNA was isolated from controls and patient fibroblasts using Pure Link™ RNA Mini Kit (Ambion). Quantitative reverse transcription polymerase chain reaction (RT-PCR) was performed in triplicate on 384-Well reaction plates using a 7900HT Fast Real-Time PCR System (Applied Biosystems). Fifty-nanogram total RNA was retro-transcribed and amplified in ‘one-step’ reactions using QuantiFast SYBR Green RT-PCR Kit (QIAGEN). Expression of 12S and 16S rRNA was normalized to expression of the housekeeping ß-actin gene (ACTB) and results were represented as fold changes in the patient relative to controls. Primer-sequences are listed in Supplementary Material, Table S3.

### Sucrose gradient sedimentation

A total of 5 × 10^6^ fibroblasts were lysed in 700 ul of lysis buffer (40 mM Tris-HCl, pH 7.6, 150 mM NaCl, 20 mM MgCl_2_, 1% n-dodecyl-D-maltoside (DDM), 1 mM PMSF, 1X protease inhibitors without EDTA, 0.08 U/mL RNasin) on ice for 45 min. Lysates were cleared by centrifugation at 12 000× g for 40 min and 1 mg lots of protein were loaded onto a 10–30% linear sucrose gradient. Gradients were prepared in 40 mM Tris-HCl, 150 mM NaCl, 20 mM MgCl_2_, 1 mM PMSF, 1X protease inhibitors without EDTA, using a gradient master (Biocomp), according to the manufacturer instructions. Samples were centrifuged at 71 000× g for 16 h in a Beckman SW41 rotor at 4°C. After centrifugation, 16 fractions of 750 μl each were collected from the top of the gradient and analysed by immunoblotting. For MRPS25 analysis, the protein content of each fraction was concentrated by trichloroacetic acid (TCA) precipitation before western blot analysis.

### Lentiviral expression of wild-type *MRPS25*

*MRPS25* cDNA clone (BC003590.2) was obtained from BioCat GmbH (Heidelberg, Germany). A MRPS25-Flag construct was generated with a pair of oligonucleotides (forward primer: 5′- Ctt ctt Gaa Ttc acc atg cccatgaagggccgcttccccatc -3′, reverse primer: 5′- ctctct aagctt cta cttatcgtcgtcatccttgtaatc gtcctgggcatcggctttca-3′), which include EcoRI and HindIII restriction sites and the Flag tag, and was inserted into pENTR vector (Life Technologies, Darmstadt, Germany). After generating an entry clone, the pLenti CMV Blast DEST MRPS25-Flag expressing vector (Addgene plasmid #17451) was created using LR recombination. The pLenti CMV Blast DEST MRPS25-Flag expressing vector and packaging plasmids pMD2.G and psPAX2 (Addgene plasmid #12259 and #12260) were transfected into HEK-293FT cells with lipofectamin 2000 (Life Technologies, ThermoFisher Scientific, Darmstadt, Germany) and OPTI-MEM media (Life Technologies, ThermoFisher Scientific, Darmstadt, Germany) to produce the lentivirus. The recombinant lentiviral constructs were transduced into the fibroblast cell lines that had been immortalized by retroviral expression of the HPV-16 E6E7 gene. Stable cell lines expressing the MRPS25-Flag protein were generated by blasticidin (Invitrogen) selection.

### Modelling of human MRPS25

Multiple sequence alignment was done using the Clustal Omega (https://www.ebi.ac.uk/Tools/msa). The structural model image was prepared with the PyMOL Molecular Graphics System, Version 1.2r3pre, Schrödinger, LLC.

### Statistics

Statistical analyses were performed using Graphpad Prism v.7.04. Immunoblots and mitochondrial protein synthesis were analyzed with Fiji ImageJ. *T*-test was used for comparing two independent groups. *P*-values <0.05 were considered to be statistically significant.

## Supplementary Material

HMG-2019-D-00137_Bugiardini_R1_Supplementary_Table_S1_ddz093

HMG-2019-D-00137_Bugiardini_R1_SUPPLEMENTARY_TABLES_2_and_3_ddz093
