# Supplementary material for: MRPS25 mutations impair mitochondrial translation and cause encephalomyopathy
Source: Hum Mol Genet. 2019 Apr 30;28(16):2711–9. doi: 10.1093/hmg/ddz093 (PMC6687946; doi:10.1093/hmg/ddz093)
Supplement: HMG-2019-D-00137_Bugiardini_R1_SUPPLEMENTARY_TABLES_2_and_3_ddz093 [file hmg-2019-d-00137_bugiardini_r1_supplementary_tables_2_and_3_ddz093.docx]

**Table S2. List of all antibodies employed for Western Blot throughout the study**

| **Antibody** | **Company** | **Catalog** ♯ | **Dilution** |
| --- | --- | --- | --- |
| anti-MPRS17 | Proteintech | 18881-1-AP | 1:1000 |
| anti-MPRS22 | Proteintech | 10984-1-AP | 1:1000 |
| anti-MPRS25 | Proteintech | 15277-1-AP | 1:1000 |
| anti- MPRS27 | Proteintech | 17280-1-AP | 1:2000 |
| anti-MRPS29 | Abcam | Ab11928 | 1:1000 |
| anti-MRPL13 | Proteintech | 16241-1-AP | 1:2000 |
| anti-MRPL44 | GeneTex | GTX121263 | 1:1000 |
| anti-MRPL45 | Proteintech | 15682-1-AP | 1:1000 |
| anti-NDUFB8 | Abcam | Ab110242 | 1:2000 |
| anti-NDUFA9 | Abcam | ab14713 | 1:2000 |
| anti-COX1 | Abcam | ab14705 | 1:2000 |
| anti-COX2 | Abcam | ab110258 | 1:2000 |
| anti-COX4 | Abcam | ab14744 | 1:2000 |
| anti-UQCRC1 | Abcam | ab110252 | 1:1000 |
| anti-UQCRC2 | Abcam | ab14745 | 1:1000 |
| anti-UQCRFS1 | Abcam | ab14746 | 1:1000 |
| anti-ATP5A | Abcam | ab14748 | 1:5000 |
| anti-VINCULIN | Abcam | Ab14714 | 1:5000 |

**Table S3. Primers used for RT-PCR**

| **Primer name** | **Sequence 5’-3’** |
| --- | --- |
| 12S-F | CCCCAGGTTGGTCAATTTC |
| 12S-R | CGGCTTCTATGACTTGGGTTAA |
| 16S-F | TTTGCAAGGAGAGCCAAAGC |
| 16S-R | AGACGGGTGTGCTCTTTTAGC |
| ACTB-F | GACGACATGGAGAAAATCT |
| ACTB-R | ATGATCTGGGTCATCTTCTC |
